# *In Vitro* Synergistic Enhancement of Newcastle Disease Virus to 5-Fluorouracil Cytotoxicity against Tumor Cells

**DOI:** 10.3390/biomedicines4010003

**Published:** 2016-01-29

**Authors:** Ahmed M. Al-Shammari, Marwa I. Salman, Yahya D. Saihood, Nahi Y. Yaseen, Khansaa Raed, Hiba Kareem Shaker, Aesar Ahmed, Aseel Khalid, Ahlam Duiach

**Affiliations:** Experimental Therapy Department, Iraqi Center for Cancer and Medical Genetic Research, Baghdad 1001, Iraq; marwa_i_salman@yahoo.ca (M.I.S.); yahya.saihood@iccmgr.org (Y.D.S.); nahiyaseen@iccmgr.org (N.Y.Y.); Khansaa.Raed@iccmgr.org (K.R.); Hiba.Kareem@iccmgr.org (H.K.S.); aesar.ahmed@iccmgr.org (A.A.); Aseel.Khalid@iccmgr.org (A.K.); Ahlam.Duiach@iccmgr.org (A.D.)

**Keywords:** NDV (Newcastle disease virus), 5-FU (5-fluorouracil), virotherapy

## Abstract

Background: Chemotherapy is one of the antitumor therapies used worldwide in spite of its serious side effects and unsatisfactory results. Many attempts have been made to increase its activity and reduce its toxicity. 5-Fluorouracil (5-FU) is still a widely-used chemotherapeutic agent, especially in combination with other chemotherapies. Combination therapy seems to be the best option for targeting tumor cells by different mechanisms. Virotherapy is a promising agent for fighting cancer because of its safety and selectivity. Newcastle disease virus is safe, and it selectively targets tumor cells. We previously demonstrated that Newcastle disease virus (NDV) could be used to augment other chemotherapeutic agents and reduce their toxicity by halving the administered dose and replacing the eliminated chemotherapeutic agents with the Newcastle disease virus; the same antitumor activity was maintained. Methods: In the current work, we tested this hypothesis on different tumor cell lines. We used the non-virulent LaSota strain of NDV in combination with 5-FU, and we measured the cytotoxicity effect. We evaluated this combination using Chou–Talalay analysis. Results: NDV was synergistic with 5-FU at low doses when used as a combination therapy on different cancer cells, and there were very mild effects on non-cancer cells. Conclusion: The combination of a virulent, non-pathogenic NDV–LaSota strain with a standard chemotherapeutic agent, 5-FU, has a synergistic effect on different tumor cells *in vitro*, suggesting this combination could be an important new adjuvant therapy for treating cancer.

## 1. Introduction

The mainstays of treatment for advanced cancers are chemotherapy and radiotherapy. However, they are limited due to the resistance of tumor cells to these agents, as well as their narrow therapeutic index. Therefore, combination therapies were invented to overcome cancer cell resistance and to increase the anti-tumor effect while considering the toxicity for normal tissue [1].

5-Fluorouracil is an important chemotherapeutic agent for many solid tumors, particularly gastrointestinal, brain, and head and neck malignancies. 5-FU has also been actively investigated during the last 40 years for many tumors. However, the role of systemic 5-FU in cancer therapy has been limited by the fact that dose-limiting side effects (myelosuppression and stomatitis) are usually reached before evidence of antitumor response [2,3]. Antitumor chemotherapeutic agents, such as 5-fluorouracil, are toxic to the small intestine and make it dysfunctional [4]. Effective antitumor strategies require a selective response between normal and tumor tissue (*i.e.*, therapeutic index). Replication component oncolytic viruses have important factors contributing to the therapeutic index by differential destruction of tumor cells with low toxicity to normal cells [5]. Combination strategies involve attacking tumor cells through different mechanisms of action, which can prevent tumor cells from having the time to develop resistance to treatment [6]. The combination of chemotherapy with virotherapy is a promising treatment approach for cancer [7]. Oncolytic virotherapy can increase the sensitivity of cancer cells to chemotherapy and radiotherapy. For example, adenoviruses are used in combination with cisplatin as an anti-osteosarcoma therapy [8], and they are used in combination with 5-FU [9] for pancreatic cancer therapy [9]. Another oncolytic virus, the Measles vaccine virus, has been used in combination with 5-FU for cholangiocarcinoma therapy [10]. Moreover, oncolytic herpes simplex virus 1 (HSV-1) expressing yeast cytosine deaminase (yCD) is a well-characterized prodrug/enzyme system that converts 5-fluorocytosine (5-FC) to 5-fluorouracil (5-FU), which can be used as an anti-tumor agent [11]. Chemovirotherapy has been evaluated in clinical trials for several chemotherapies and oncolytic viruses [12].

Newcastle disease virus (NDV) is a very promising, safe, oncolytic agent that can selectively replicate and destroy tumor cells [13]. NDVs kill tumor cells through three mechanisms that depend on the degree of virus replication and virulence. The first mechanism is by cytolysis secondary to viral replication [14]. The second mechanism involves inducing apoptosis [15,16]. Furthermore, NDV is reported to induce antigenic modifications to the tumor cells surface, making them more recognizable by the immune system [17,18]. We previously demonstrated that a virulent strain of NDV, LaSota, could be augmented and synergistically work with methotrexate to kill different tumor cell lines [19].

The aim of the present work is to examine the cytotoxicity of Newcastle disease virus in combination with 5-FU against cancer cells. The current work compared the antitumor effect of the combination of NDV with a reduced dose of chemotherapeutic agent (5-FU) to study the possibility of synergism, which may increase the potency of 5-FU antitumor activity and reduce the toxic effect of high-dose 5-FU without decreasing the antitumor activity.

## 2. Experimental Section

### 2.1. Study Design

The primary objectives of this study were to determine if the NDV LaSota strain could augment the anti-tumor effect of 5-FU. In addition, we aimed to reduce the toxicity of cancer chemotherapeutic agents by reducing the administered dose to 50%—the therapeutic dose of NDV. Later, we compared the effect of this combination therapy with the usual therapeutic dose of the chemotherapeutic agent *versus* virotherapy, alone, using Chou–Talalay analysis.

### 2.2. Cell Lines and Culture

The human Hep-2 (larynx carcinoma), RD (Rhabdomyosarcoma), and Vero cell lines were obtained from the Iraqi Center for Cancer and Medical Genetic Research (ICCMGR) Cell Bank Unit and maintained in minimum essential media (MEM) (Sigma-Aldrich, Taufkirchen, Germany) supplemented with 5% calf bovine serum (Sigma-Aldrich, Taufkirchen, Germany), 100 units/mL penicillin, and 100 µg/mL streptomycin. The AMN3 (murine mammary adenocarcinoma) cell line was maintained in RPMI-1640 (Sigma-Aldrich, Taufkirchen, Germany) supplemented with 5% calf bovine serum, 100 units/mL penicillin, and 100 µg/mL streptomycin. Cells were passaged using Trypsin-EDTA (USbiological, Salem, MA, USA), reseeded at 50% confluence twice a week, and incubated at 37 °C.

### 2.3. Virus

The lentogenic virulent strain of NDV (LaSota) was obtained from Al-Kindy Company for veterinarian vaccines (Baghdad, Iraq). A stock of infectious virus was propagated in embryonated chicken eggs, harvested from allantoic fluid, and purified from debris by centrifugation (3000 rpm, 30 min, 4 °C). NDV was quantified using a hemagglutination test in which one hemagglutination unit (HAU) is defined as the smallest virus concentration leading to visible chicken erythrocyte agglutination.

### 2.4. Chemotherapeutic Agent

5-FU (5-Fluorouracil)-SP Pharmaceuticals (Albuquerque, NM, USA) were purchased from the Radiation and Atomic Medicine Hospital (Baghdad, Iraq). This agent was diluted with a medium without calf bovine serum just before use for *in vitro* studies.

### 2.5. Combination Cytotoxicity Assays

To determine the cytotoxic effect of NDV and 5-FU in combination treatment, the MTT cell viability assay was conducted on 96-well plates (Becton, Dickinson, Franklin Lakes, NJ, USA). Hep-2, RD, AMN3, and Vero cells were seeded at 1 × 10^4^ cells/well. After 24 h. or a confluent monolayer was achieved, cells were treated with the virus alone (infected with NDV at 128 HAU with two-fold serial dilutions), the drug alone (the chemotherapeutic agent 5-FU at 5 µg in two-fold serial dilutions to 0.039 µg/mL), or a combination of the two (virus + 5-FU in two-fold serial dilutions). The procedure of adding these therapeutic agents involved addition of the virus for 2 h at room temperature to allow for viral attachment and penetration. Afterwards, cells were washed with PBS and serial dilutions of the drug were added to the infected and non-infected cells. Cell viability was measured after 72 h of infection by removing the medium, adding 28 µL of 2 mg/mL solution of MTT (Sigma-Aldrich, St. Louis, MO, USA) and incubating the cells for 1.5 h at 37 °C. After removing the MTT solution, the crystals remaining in the wells were solubilized by the addition of 130 µL of DMSO (Dimethyl Sulphoxide) (BDH, London, UK) followed by 37 °C incubation for 15 min with shaking [20]. The absorbency was determined on a microplate reader (Organon Teknika Reader 230S, Salzburg, Austria) at 492 nm (test wavelength); the assay was performed in triplicate. The inhibition rate of cell growth (the percentage of cytotoxicity) was calculated as (A − B)/A × 100, where A is the mean optical density of untreated wells, and B is the optical density of treated wells. The LC50 is the lowest concentration that kills 50% of the cells [21]. Each experiment was repeated at least three times in triplicate.

### 2.6. Chou–Talalay Analysis

The median effect doses (ED50) were calculated for the drug and NDV for each cell line. For synergism determination, NDV and 5-FU were studied as a non-constant ratio. To analyze the combination of NDV and 5-FU, Chou–Talalay combination indices (CI) were calculated using CompuSyn software (Combo Syn, Inc., Paramus, NJ, USA). Non-fixed ratios of NDV and chemotherapeutics, as well as mutually exclusive equations, were used to determine the CIs. A *CI* between 0.9 and 1.1 is considered additive, whereas *CI* < 0.9 and *CI* > 1.1 indicate synergism and antagonism, respectively [22,23].

## 3. Results

### Combination Chemotherapy and Viral Cytotoxicity in Vitro

To study the potential interaction between NDV and chemotherapy *in vitro*, the effectiveness of the combined treatment for several concentrations of 5-FU with NDV at various hemagglutination conditions was evaluated in the Hep-2, RD, AMN3, and Vero cell lines. Cells were treated with NDV and with 5-FU or with the combination of NDV and 5-FU. The cell viability was determined after 72 h using the MTT assay.

Enhanced cytotoxicity was observed for the combination of NDV and 5-FU at most doses.

In Hep-2, a laryngeal carcinoma cell line, combination treatment resulted in 76.5% growth inhibition (G.I.), and the proliferation rate (PR = 23.5%) was statistically significant (*p*: 0.0001) at a concentration of 0.625 µg 5-FU and 16 HAU, whereas NDV alone had 67.5% G.I. (PR = 32.5%) (*p*: 0.0001) at 16 HAU. 5-FU at 1.25 µg/mL (two times concentration) produced 73% growth inhibition (*p*: 0.0001), and no significant differences were observed between the combination therapy and 5-FU or virus therapy.

**Figure 1:**
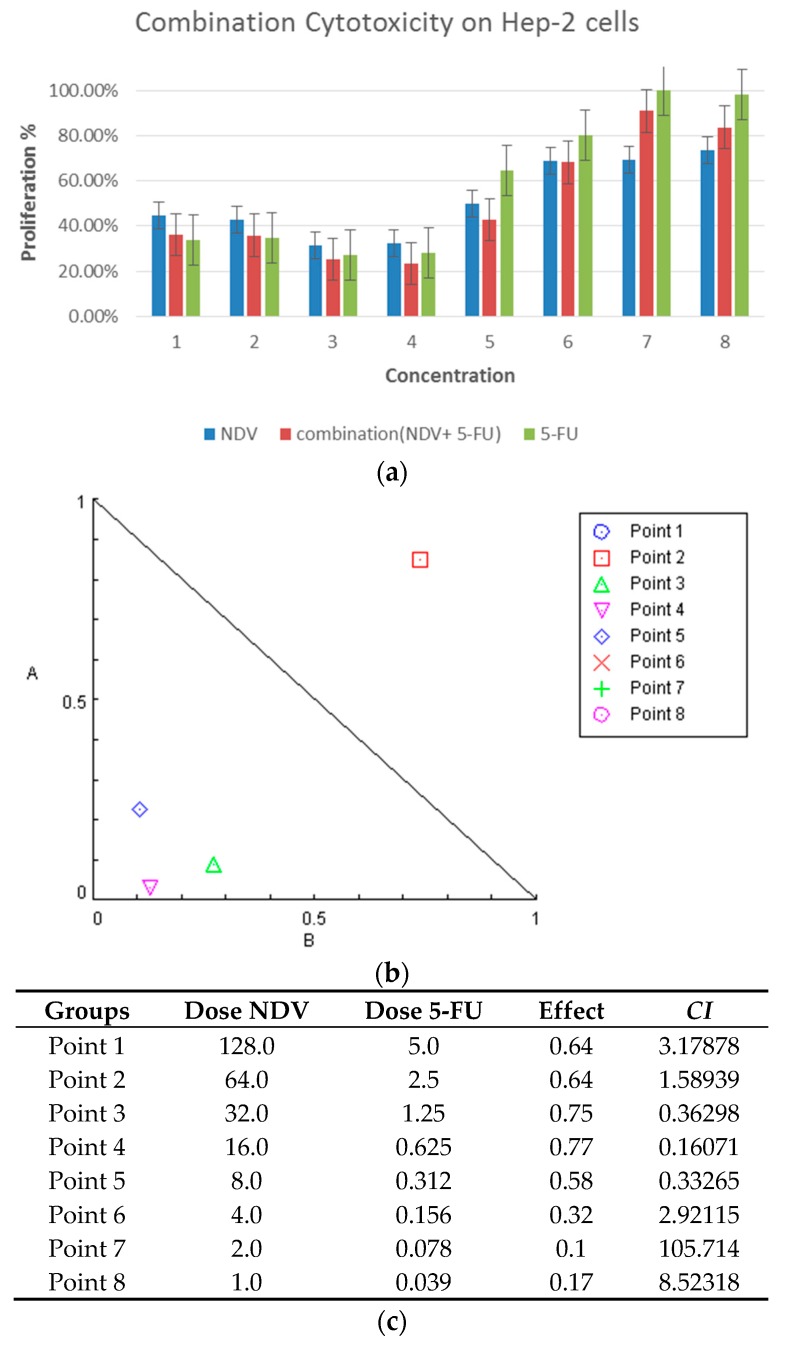
Cytotoxicity of the combination on Hep-2 laryngeal carcinoma cells. (**a**) Cytotoxic effect of 5-FU, NDV, or both, on Hep-2 cells *in vitro*. Hep-2 cells were treated with 5-FU (5 µg in two-fold serial dilutions up to 0.039 µg/mL), NDV (128 HUA with two-fold serial dilutions), or a combination of the two treatments. The results for the treated groups are expressed as the cell proliferation rate compared with untreated control cells grown at identical conditions. HUA: hemagglutination unit, which is the smallest virus concentration leading to visible erythrocyte agglutination; (**b**) isobologram analysis showing synergism between NDV and 5-FU as the point of combination is at the lower left of the hypotenuse, indicating the effect is synergistic at a 50% growth inhibition dose for the Hep-2 tumor cell line. In the figure we can see four points that is less than or equal to 1; and (**c**) table showing the combination index data for each dose.

Combination therapy at a concentration of 0.312 µg/mL and 8 HAU had 57.2% G.I. (PR = 42.8%) (*p*: 0.0001). NDV therapy alone had 50% G.I. (PR = 50%) (*p*: 0.002), while 5-FU alone at two-fold the dose (0.625 µg/mL) has only 35.5% growth inhibition (PR = 64.5%), which is not significant (*p*: 0.018), as shown in Figure 1a.

Potential interactions between NDV and anti-cancer 5-FU were evaluated using Chou–Talalay equations. A *CI* < 0.9 is considered synergistic, *CI* between 0.9 and 1.1 is considered additive, and *CI* > 1.1 is considered antagonistic [22]. Using the dose-oriented isobologram technique, the Hep-2 laryngeal carcinoma cell line had synergism between NDV and 5-FU at 50% growth inhibition doses, as shown in (Figure 1b), which explain the synergism effect at combination points 3 (*CI*: 0.36298), 4 (*CI*: 0.16071), and 5 (*CI*: 0.33265).

**Figure 2:**
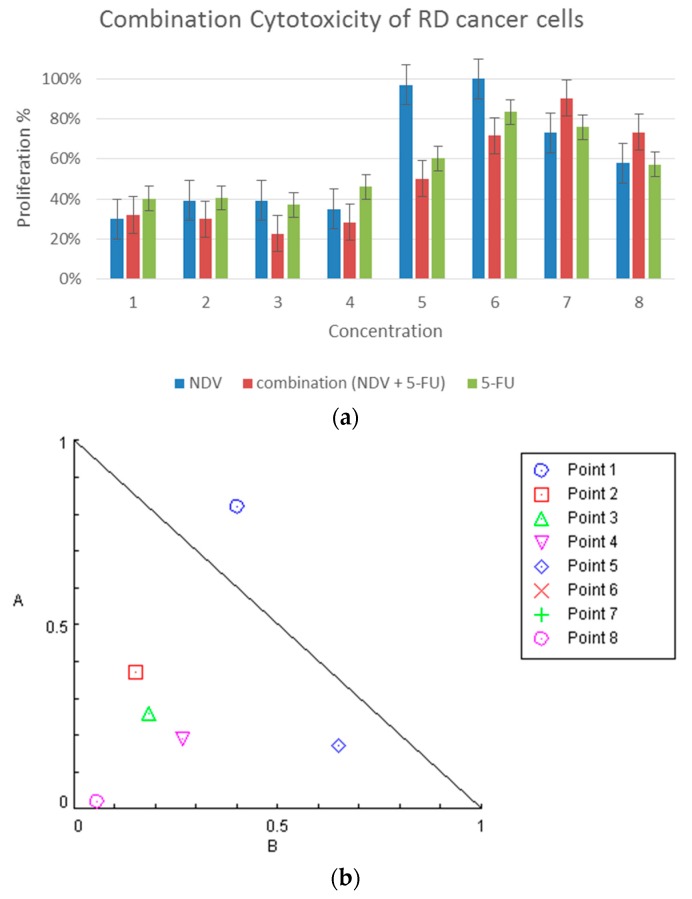

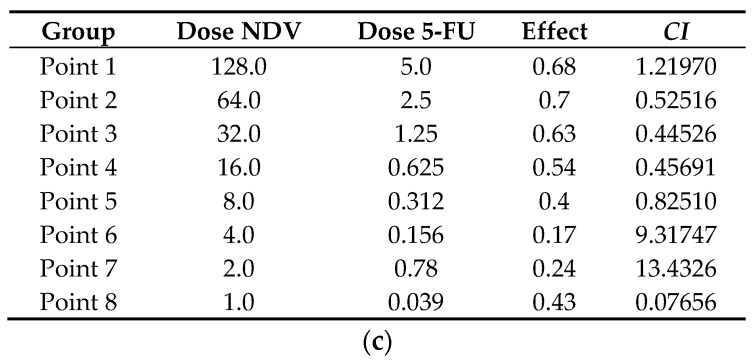
Combination cytotoxicity on RD cells. (**a**) Cytotoxic effect of 5-FU, NDV, or both, on RD cell proliferation. The cells were treated with 5-FU (5 µg in two-fold serial dilutions reaching 0.039 µg/mL), NDV (128 HUA with two-fold serial dilutions), or a combination of the two. The results for the treated groups are expressed as the cell proliferation rate compared with untreated control cells grown under identical conditions; (**b**) isobologram analysis showing synergism between NDV and 5-FU at the most higher doses and antagonism at lower doses (while the points that are extremely antagonized will not show in the figure such as point 6 and 7 as their value above 2 and will not be able to be included) ; and (**c**) table summarizing the combination index data for each dose of combination points.

In RD, Rhabdomyosarcoma, combination therapy had significant 71.6% cytotoxicity (PR = 29%) (*p*: 0.0001) at 0.625 µg/mL 5-FU and 16 HAU NDV. NDV treatment alone showed 64.1% cytotoxicity (PR = 35.9%) (*p*: 0.0001) at 16 HAU, and 1.25 µg/mL 5-FU therapy alone also showed a significant cytotoxic effect of 53.8% (PR = 46.2%) (*p*: 0.0001), which is less than the combination of NDV and half the dose of 5-FU. Combination therapy at 0.312 µg/mL 5-FU and 8HAU showed 49.7% G.I. (*p*: 0.002), whereas the growth inhibition effect of chemotherapy alone with 5-FU at two-fold dose (0.625 µg/mL) was 39.7% G.I. (PR = 60.3%) (*p*: 0.1) (Figure 2a). Data were further analyzed using Chou–Talalay equations and the dose-oriented isobologram technique. There was synergism between NDV and 5-FU at 50% growth inhibition doses, as represented in Figure 2b, which explains the synergism effect at the combination points 2 (*CI*: 0.52516), 3 (*CI*: 0.44526), 4 (*CI*: 0.45691) and 5 (*CI*: 0.82510). The combination point dose details are described in Figure 2c.

**Figure 3:**
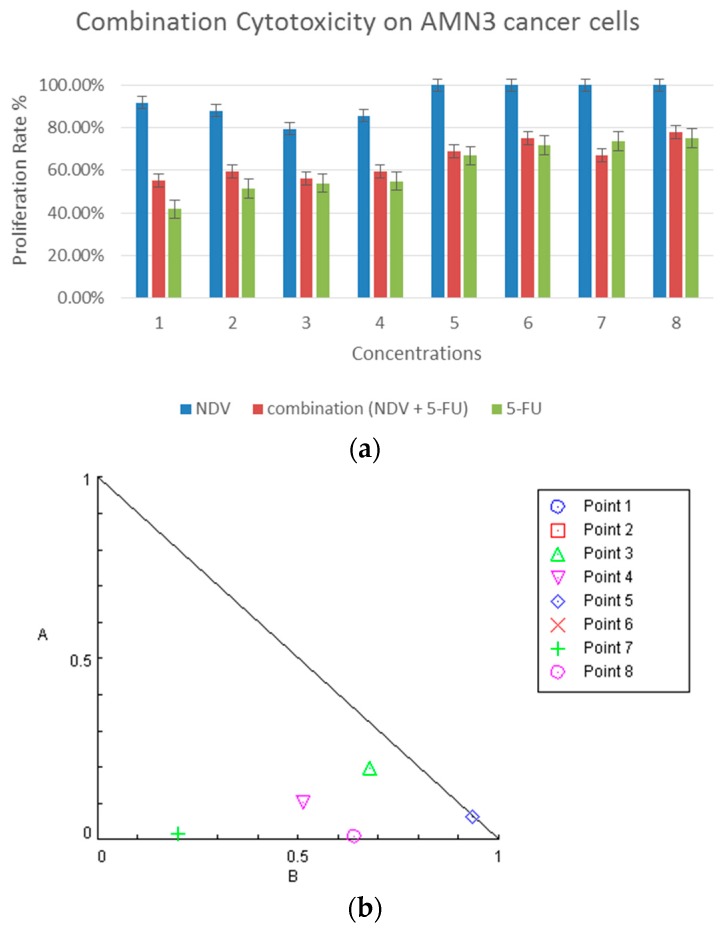

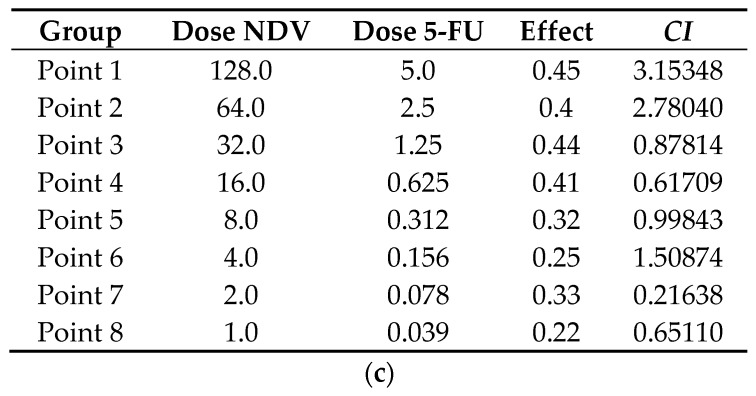
Combination cytotoxicity on AMN3 cells. (**a**) The cytotoxic effect of 5-FU, NDV, or both, on AMN3 cell proliferation. The cells were treated with 5-FU (5 µg in two-fold serial dilutions up to 0.039 µg/mL), NDV (128 HUA with two-fold serial dilutions) or a combination of the two. The results for the treated groups are expressed as the cell proliferation rate compared to untreated control cells grown at identical conditions; (**b**) isobologram analysis shows the synergism between NDV and 5-FU at the highest five doses and antagonism at three doses (point 1 and 2 value above 2 and for that will not be shown in the figure limits as they considered as extremely antagonized); and (**c**) table showing the combination index data for each dose of the combination points used.

In AMN3, mouse mammary adenocarcinoma cell line combination therapy was effective (*p*: 0.006) and had a similar effect as with 5-FU alone at two-fold doses. There were no significant differences between combination with half-dose of chemotherapy compared with 5-FU alone. These results show that NDV could compensate for the reduction in the 5-FU doses even though there was no significant effect of NDV treatment on tumor cells alone at any of the concentration (Figure 3a). Further analysis using the Chou–Talalay equation and dose-oriented isobologram technique analysis showed synergism between NDV and 5-FU in five combinations, points 3 (*CI*: 0.87814), 4 (*CI*: 0.61709), 5 (*CI*: 0.99843), and 7 (*CI*: 0.21638), at 50% growth inhibition doses, as represented in Figure 3b,c.

**Figure 4:**
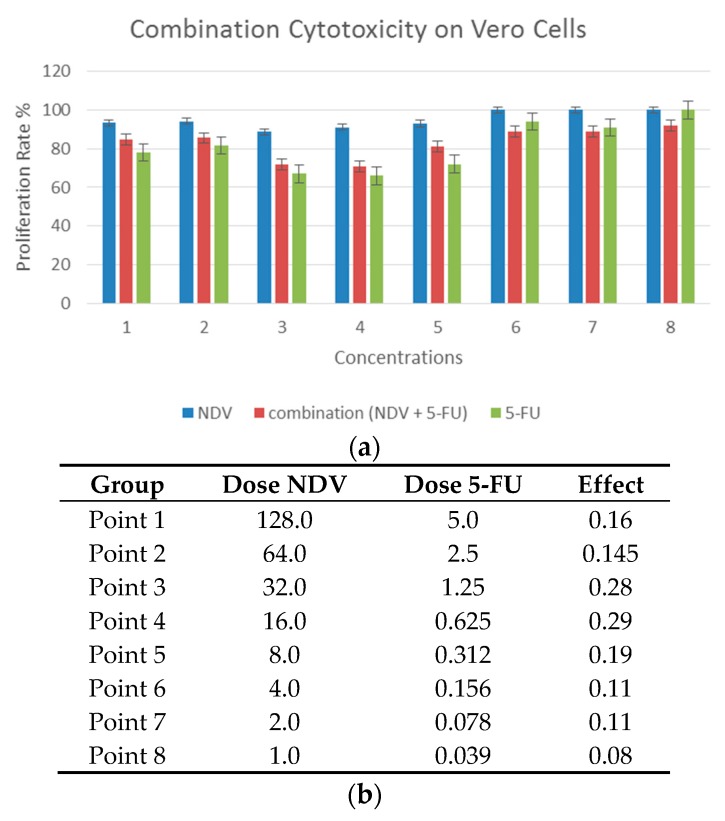
Combination cytotoxicity on Monkey kidney Vero cells. (**a**) The cytotoxic effect of 5-FU, NDV, or both, on cell proliferation. The cells were treated with 5-FU (5 µg in two-fold serial dilutions up to 0.039 µg/mL), NDV (128 HUA with two-fold serial dilutions) or a combination of the two. The results for the treated groups are expressed as the cell proliferation rate compared with untreated control cells grown under identical conditions. Most of the concentrations used alone or in combination treatment lacked significant differences; and (**b**) table summarizing the combination data for each dose of combination points.

To study the effect of combination treatment on non-cancer cells, the Vero monkey kidney transformed cell line was used. Generally, most of the concentrations used alone or in combination lacked significant differences (Figure 4). As there was no significant cytotoxic effect, there was no need to do Chou–Talalay equation.

## 4. Discussion

The primary objective of this study was to determine if we can augment cancer chemotherapy by virotherapy. Furthermore, we sought to determine if we can reduce the toxicity of cancer chemotherapeutic agents by reducing the administered dose of 5-FU and replacing it with Newcastle disease virus therapy, while maintaining the same, or more, anti-tumor activity and overcoming resistance to chemotherapy.

Based on our results in four different cell lines, the lentogenic NDV strain (LaSota used as live vaccines against NDV) exhibited oncolytic activity on three tumor cell lines and to a lower degree on the transformed cell line (Vero). Previous studies have shown that a virulent NDV strain is oncolytic [14,24]. The NDV LaSota strain showed anti-lymphoma activity both *in vitro* and *in vivo* [25]. Furthermore, Walter *et al.* [26] showed that the Newcastle Disease Virus LaSota strain kills human pancreatic cancer cells *in vitro* with 700-fold higher selectivity than normal cells.

Fabian *et al.* [27] used an attenuated pathogenic NDV MTH-68/H strain, which was originally a vaccine strain. This strain showed antitumor activity both *in vitro* and *in vivo* in a clinical trial [28,29]. Moreover, Pecorna *et al.* [30] used a naturally attenuated strain of NDV (PV701) that exhibits a broad range of oncolytic activity against human tumors *in vitro*; they introduced the strain into clinical trials. Schirrmacher *et al.* [14] used a lentogenic virulent Ulster strain and found that infection of cancer cells by non-lytic non-virulent NDV Ulster strain (30 HU/107 cells) eventually causes tumor cell death *in vitro*, and it also has replication selectivity in tumor cells [31].

The combination of NDV and 5-FU showed greater cytotoxic efficacy than NDV alone or at a two-fold dose of 5-FU alone. The effect appears to be synergistic according to Chou–Talalay analysis. The mechanism(s) of synergistic activity for the combination of 5-FU with NDV is unknown, but we propose a few hypotheses. First, NDV may augment the anti-tumor activity of 5-FU by increasing the cellular sensitivity to chemotherapeutic agents, and this enhanced sensitivity is partially caused by the induction of apoptosis in response to virulent NDV strains [32]. Second, a synergistic dose of 5-FU may augment the viral replication, as suggested by many studies on oncolytic viruses [19,33]. Each agent may work independently on different cell populations, but this is unlikely to be the case here. In addition, virotherapy with NDV may complement the anti-tumor activity of 5-FU, which selectively targets tumor cell populations that are resistant to chemotherapy. This may be of important value because most human tumors consist of a mixture of cells that have a different genetic makeup. Heterogeneity in the tumor cell populations may be the major reason most monotherapies fail to achieve complete tumor remission [34]. Moreover, one of the objectives of this study was to reduce the toxic side effects of chemotherapy in cancer patients. This can be achieved by reducing the administered dose while maintaining the same or stronger antitumor activity. The current experimental results support this claim, but *in vivo* evaluation is needed.

Several characteristics of the NDV LaSota strain that are favorable for human use include the genetic stability of the vaccine strains, absence of genetic recombination, lack of antigenic drift, and lack of observed human to human transmission [35,36]. The Newcastle disease virus has been safely administered to humans in clinical trials; additionally, accidental exposure in farmers is reported to induce only self-limiting conjunctivitis [28,35,37]. While NDV is safe and lacks toxicity, 5-FU causes myelosuppression and stomatitis before achieving an antitumor response [2].

## 5. Conclusions

A virulent, non-pathogenic NDV LaSota strain, in combination with a standard chemotherapeutic agent, 5-FU, has a synergistic effect *in vitro* on different tumor cells, suggesting this approach could be an important new adjuvant therapy for treating cancer.
